# The Effect of Group-Based Early Parent Education on Development in Preterm Infants

**DOI:** 10.3390/children11121461

**Published:** 2024-11-29

**Authors:** Joon Hee Lee, Nam Hyun Lee, In Jin Yoon, Mi Jin Hong, Eun Jung Choi, Baek Hee Jang, Jong Yoon Chang, Byong Sop Lee, Euiseok Jung, In Young Sung, Eun Jae Ko

**Affiliations:** 1 Department of Rehabilitation Medicine, Asan Medical Center, College of Medicine, University of Ulsan, Seoul 05505, Republic of Korea; fredjh@naver.com (J.H.L.); fevernova31@naver.com (B.H.J.); changshine@naver.com (J.Y.C.); 2 Department of Rehabilitation Medicine, Asan Medical Center, Seoul 05505, Republic of Korea; rptlee@amc.seoul.kr (N.H.L.);; 3 Department of Rehabilitation Medicine, Konyang Medical Center, College of Medicine, University of Konyang, Daejeon 35365, Republic of Korea; 200612@kyuh.ac.kr; 4 Department of Rehabilitation Medicine, Seongnam Citizens Medical Center, Seongnam 13290, Republic of Korea; mttly@scmc.kr; 5 Department of Pediatrics, Asan Medical Center Children’s Hospital, College of Medicine, University of Ulsan, Seoul 05505, Republic of Korea; mdleebs@amc.seoul.kr (B.S.L.); euisjung@amc.seoul.kr (E.J.)

**Keywords:** group-based education, parent education, preterm infants

## Abstract

Objectives: This study explored how group-based early parent education impacts development in preterm infants. Methods: This retrospective cohort study, with a historical control group, included preterm infants (n = 246) with corrected ages (CAs) of 0–3 months. Those visiting the clinic between July 2017 and December 2018 formed the control group (n = 145), whereas those visiting between January 2019 and February 2020 (n = 101) constituted the education group. The education group received six education sessions performed by a physical therapist, and two sessions conducted by an occupational therapist at CAs of 0–6 months. Assessments included the following: Alberta Infant Motor Scale at CA 3, 6, 9, and 12 months; Bayley Scales of Infant Development II at CA 12 and 24 months; and the Sequenced Language Scale for Infants at CA 24 months. Results: The education group, which initially showed greater developmental delay than the control group, showed no significant delay after education sessions. Developmental improvements were more prominent in infants born before 28 weeks’ gestational age, and in infants with no, or mild, brain injury. Conclusions: Group-based early parent education improved development at CA 24 months in preterm infants, especially in infants born before 28 weeks gestational age, and in infants with no, or mild, brain injury. This approach may enhance developmental outcomes in preterm infants.

## 1. Introduction

Preterm birth, before 37 weeks of complete gestation, is associated with increased mortality and long-term morbidity. The survival of preterm infants is improving due to the progress of neonatal intensive care unit (NICU) care and other treatments, such as antenatal corticosteroid administration. Many studies indicate that the survival of preterm infants exceeds predicted rates [1,2]. However, a study comparing two birth cohorts between 1995 and 2006 showed that, although the survival rate of extremely preterm-birth infants increased, the pattern of major neonatal morbidities, such as bronchopulmonary dysplasia, remained unchanged. Among these morbidities, cerebral injuries, such as ventriculomegaly, hemorrhagic parenchymal infarction, or parenchymal cysts, were the major factors [3]. Neurodevelopmental impairment is one of the abnormalities that can follow preterm birth, causing severe disabilities in children as they grow up. Preterm infants exhibit developmental delays, including in motor skills, cognition, and behavior [4]. Additionally, the risk of cerebral palsy is higher in preterm infants and increases as gestational age at delivery decreases [5].

The developing brains of preterm infants during the early stages of life exhibit considerable plasticity, suggesting excellent developmental potential [6]. The dysregulation of neuroplasticity in preterm infants can result in impaired cerebral maturation, leading to disabilities [7]. To minimize these disabilities and achieve optimal brain development, early developmental interventions have been used and have improved infants’ cognitive and behavioral outcomes. One intervention, which focused on improving infant–parent interaction, comprised an initial debriefing session for parents followed by a 1 h session for infants and parents addressing how to respond to infants’ signs and develop mutual social interaction [8]. A recent study also demonstrated that parent-guided developmental intervention on preterm infants improved infant neurodevelopmental outcomes [9]. Another study implemented a program focused on instructing parents to provide tactile/kinesthetic stimulation. The program also included home-based intervention and assessed parents’ comprehension and compliance to ensure active participation. A recent systematic review also showed that many environmental strategies promoted development in both cognitive and motor domains [10].

Beyond the direct rehabilitation of preterm infants, the interplay between developmental capacity and environmental factors contributes to outcomes [11]. As a critical environmental factor, caregiver behavior, specifically parenting style, is associated with developmental outcomes [12,13]. Various types of early interventions, each with different timing and contents focused on families and communities, are employed to improve infant development. A meta-analysis of family-focused early intervention demonstrated positive effects on preterm infants, particularly regarding cognition [14].

At our hospital, we implemented an education program comprising eight group sessions. The program differs from previous studies because it emphasizes group-based education, starting very early at the corrected age (CA) 0–3 months. Our program also integrates both physical therapy and occupational therapy techniques. The purpose of the program was to provide maximal stimulation in various domains including sensory experiences. Most previous studies and programs focused on physical therapy techniques. However, a recent study demonstrated the potential of occupational therapy intervention on the development of preterm infants [15]. In this context, the present study aimed to evaluate the effectiveness of this unique early group-based parent education system in the development of preterm infants.

## 2. Materials and Methods

### 2.1. Study Design and Participants

This was a retrospective cohort study with historic controls. The study was approved by the hospital’s Institutional Review Board (No. 2023-0038) and received an exemption for consent as a retrospective chart review. Preterm infants of CA 0–3 months who were born at the same tertiary hospital, admitted in the same neonatal unit, and those who visited the outpatient clinic for pediatric rehabilitation medicine, were included. Infants who visited the clinic from July 2017 to December 2018 were assigned to the control group, and children who visited the clinic from January 2019 to February 2020 were assigned to the education group. Children who could not participate in the rehabilitation program due to a poor medical condition or living at distance, and those without sufficient data in the electronic medical record system, were excluded.

### 2.2. Intervention

Both the education and control groups received identical standard medical care. The infants also went through standard rehabilitation sessions when they showed evidence of developmental delay, brain image abnormality, or neurologic abnormality such as abnormal muscle tone. The standard rehabilitation sessions included bedside physical therapy at the NICU, an outpatient rehabilitation program, or an intensive inpatient rehabilitation program, when needed.

The education group received eight additional sessions of a group-based parent education program. The education was delivered to the whole group together (up to four infants and four parents per group) and consisted of 30 min of education per session. These sessions included four sessions during the CA 0- to 3-month period and four sessions during the CA 3- to 6-month period. During each period, two experienced physical therapists and two experienced occupational therapists offered three sessions of education related to physical therapy and one session of education related to occupational therapy. The activities educated in the sessions are described in Table 1. The therapists demonstrated activities in front of the group of infants and parents, and encouraged each parent to perform the activities that they learned with the infant. If the parent was inexperienced in doing the activity or had any questions, the therapist provided additional 1:1 guidance and feedback throughout the session. The parents were encouraged to administer activities at their home three times a day. There was no questionnaire regarding how many times the parent performed the activities at home, but when verbally asked, most of the parents answered that they performed the activities with their infants at least twice a day.

### 2.3. Measurements

**Figure 1:**
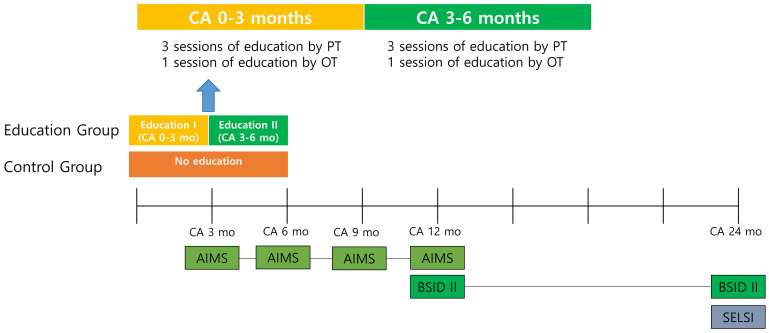
Timeline of group-based parent education program and development assessment.

All infants participating in the study underwent standardized assessments at CA 3, 6, 9, 12, and 24 months. These assessments were conducted by two physical therapists and two occupational therapists who were not involved in implementing the intervention. The Alberta Infant Motor Scale (AIMS) was used to evaluate motor development at CA 3, 6, 9, and 12 months. For the assessment of cognitive and motor development, the Bayley Scale of Infant Development II (BSID-II) was used at CA 12 and 24 months. The Sequenced Language Scale for Infants (SELSI) was used to assess language development at CA 24 months. The primary study outcome was a BSID-II score at CA 24 months, whereas the secondary outcomes were the remaining measurements. The timeline of the study is demonstrated in Figure 1.

The BSID-II is widely used to assess developmental progress and is considered the best measure for infants and toddlers aged 1 –42 months, with high validity and reliability [16,17]. Raw scores from the BSID-II are converted to the Mental Development Index (MDI) for the cognitive scale and to the Psychomotor Developmental Scale (PDI) for the motor scale. The cut-off values of 85 and 70 are used to discriminate mild and moderate-to-severe developmental delays, respectively [18].

The AIMS examines motor development from term until 18 months of age [19]. It comprises 58 items, categorized into four subscales: prone (21 items), supine (9 items), sitting (12 items), and standing (16 items). The test’s validity and interobserver reliability have been verified [20]. Research by Pin et al. [21] suggests that the AIMS is a sensitive assessment tool for capturing the movement characteristics of preterm infants. The raw score of the AIMS was used in this study.

The SELSI evaluates receptive and expressive language abilities. Raw scores are then converted into the Receptive Language Index (RLI) and Expressive Language Index (ELI). The SELSI was proven to be valid and reliable through previous study, with language delay defined as having a language age more than two standard deviations below corresponding ages on the RLI and ELI [22,23].

In addition, data were collected on baseline characteristics, including gestational age (extremely preterm: <28 weeks; very preterm: 28 to <32 weeks; moderate-to-late preterm: 32 to <37 weeks), birthweight, sex, presence and severity of brain lesions (no abnormal findings; mild lesion: intraventricular hemorrhage (IVH) grade 1 or 2; moderate-to-severe lesion: IVH grade 3 or 4, or periventricular leukomalacia (PVL); others, e.g., stroke, ventriculomegaly, tubulinopathy), delivery method, complications such as retinopathy of prematurity (ROP), necrotizing enterocolitis (NEC), history of seizures, major surgeries (such as open abdominal/thoracic surgery or laparoscopic/thoracoscopic surgery), and genetic anomalies and cerebral palsy.

### 2.4. Statistical Analyses

Statistical analyses were conducted using SAS software version 9.4 (SAS Institute Inc.; Cary, NC, USA). To compare baseline characteristics, independent *t*-tests or Mann–Whitney U-tests were conducted for continuous variables, and Pearson’s Chi-squared test was performed for categoric variables. Additionally, for developmental outcomes between the two groups, an independent *t*-test or Mann–Whitney U-test was used for continuous variables. Further, the effect of the group-based parent education was analyzed based on gestational age and brain lesions.

## 3. Results

### 3.1. Baseline Characteristics

When comparing baseline characteristics between the two groups, the education group had a higher proportion of infants with a lower gestational age, more severe brain abnormalities, and greater surfactant use (Table 2).

### 3.2. Developmental Outcomes

When comparing initial development in the two groups, the education group showed a significant delay in AIMS at 3 and 6 months compared to the control group. However, the education group had a significantly higher AIMS score at 9 months. Additionally, there were no significant between-group differences in the developmental scores at 12 and 24 months regarding AIMS, MDI, and PDI of BSID-II, and RLI and ELI of SELSI. The education group had a greater number of infants diagnosed with cerebral palsy (Table 3).

### 3.3. Developmental Outcomes According to Gestational Age

**Figure 2:**
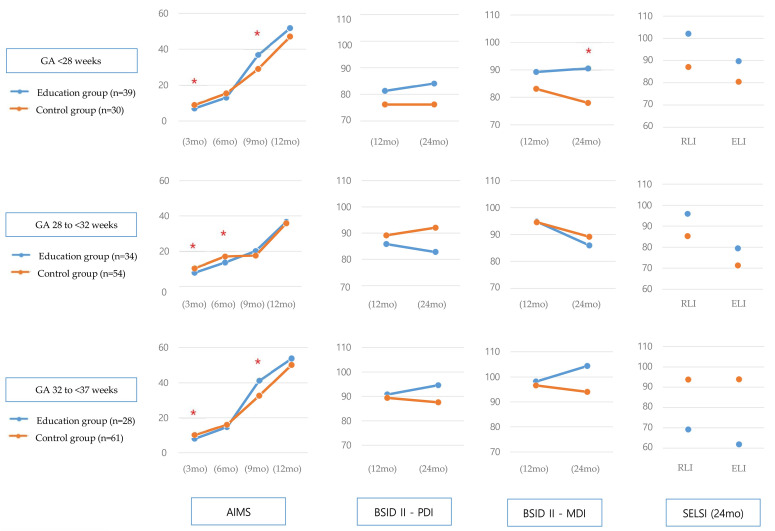
Development outcomes according to gestational age. * *p* < 0.05 by independent *t*-test or Mann–Whitney U-test. Abbreviations: AIMS, Alberta Infant Motor Scale; BSID II, Bayley Scale of Infant Development II; ELI, Expressive Language Index; GA, Gestational Age; MDI, Mental Development Index; PDI, Psychomotor Development Index; RLI, Receptive Language Index; SELSI, Sequenced Language Scale for Infants.

The education group versus control group had a significant delay in the AIMS score at 3 months in all three age groups: infants born earlier than 28 weeks, infants born between 28 and 32 weeks, and infants born between 32 and 37 weeks. However, after CA 9 months, the education group had a higher AIMS score; at 9 months, the differences were statistically significant in infants born earlier than 28 weeks, and in infants born between 32 and 37 weeks. After 12 months, the MDI and PDI scores of BSID II and the RLI and ELI scores of SELSI in the education group were generally higher than in the control group. In addition, the MDI score of BSID II at 24 months in infants born earlier than 28 weeks showed a significantly higher developmental status for the education group than the control group (Figure 2, Appendix A).

### 3.4. Development Outcomes According to Brain Injury

**Figure 3:**
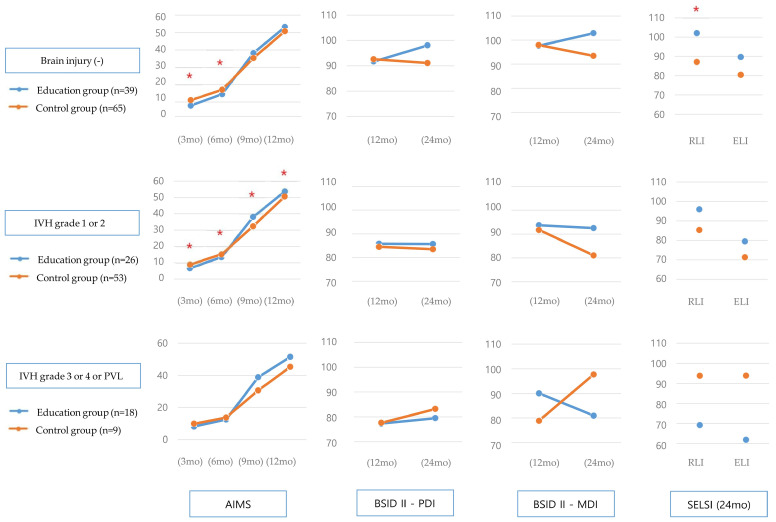
Development outcomes according to brain injury. * *p* < 0.05 by independent *t*-test or Mann–Whitney U-test. Abbreviations: AIMS, Alberta Infant Motor Scale; BSID II, Bayley Scale of Infant Development II; ELI, Expressive Language Index; IVH, intraventricular hemorrhage; MDI, Mental Development Index; PDI, Psychomotor Development Index; PVL, periventricular leukomalacia; RLI, Receptive Language Index; SELSI, Sequenced Language Scale for Infants.

The education group versus control group showed a tendency towards delay in the AIMS score at 3 months in all three brain injury groups: infants born without brain abnormality, infants with mild abnormalities (IVH grade 1 or 2), and infants with moderate-to-severe abnormalities (IVH grade 3 or 4, or PVL). However, after CA 9 months, the education group showed a tendency towards higher AIMS scores; at 9 months, the between-group difference was statistically significant in infants with mild abnormalities. After 12 months, the MDI and PDI scores of BSID II and the RLI and ELI scores of SELSI in the education group (no and mild brain injury) were higher than in the control group. Further, the RLI score of SELSI at 24 months in infants without brain abnormalities showed significantly higher developmental status for the education versus control group (Figure 3, Appendix A).

## 4. Discussion

This study demonstrates that the eight sessions of a group-based early parent education program for preterm infants led to improved development over 24 months, even when the infants initially had delayed development at CA 3 months. The intervention showed a greater improvement for preterm infants born earlier than 28 weeks, as evidenced by the MDI score of BSID-II at 24 months and a significant improvement in infants with mild-to-moderate brain lesions (IVH grade 1 or 2), as indicated by the AIMS scores at 6, 9, and 12 months. Infants without recognized brain lesions showed significant improvement in the RLI score of SELSI at 24 months.

Although all infants showed improvements regarding their motor scores and cognition/language abilities, the effect of education was more pronounced in preterm infants born before 28 weeks. Specifically, the lower developmental scores observed at 3 months significantly reversed in favor of the education group, as evidenced by the MDI score of BSID-II at 24 months. These results suggest that the program was more helpful for high-risk preterm infants. One possible explanation is that preterm infants born after 28 weeks may already perform well developmentally. A meta-analysis of cognitive and behavioral outcomes in preterm children demonstrated a correlation between low gestational age and decreased cognitive function scores [24]. Another recent study on association between gestational age at birth and development showed that gestational age was inversely associated with developmental delays [25]. These imply that relatively well-developed preterm infants born after 28 weeks leave limited room for improvement related to environmental intervention, whereas infants born before 28 weeks have greater room for improvement. In our study, the greater improvement in the education group compared to the control group suggests that improvement was due primarily to education.

Subgroup analyses, considering the presence and severity of brain abnormalities, showed that the effect of education was more pronounced in preterm infants with mild-to-moderate brain injury. Although the target disease entity and age differed from our study, a study on predicting recovery from head injury in children showed that injury severity determined by radiological findings correlated with worse prognosis after brain injury [26]. Another study also demonstrated that the increasing severity of IVH is associated with higher risk of adverse outcomes [27]. Our results suggest that infants with definite brain abnormalities—such as IVH grade 3 or 4, or PVL, as identified through radiographic measures—retain a relatively lower level of neuroplasticity, leading to low developmental potential, despite early parent education. In contrast, preterm infants with no or mild brain injury (such as IVH grade 1 or 2) may exhibit substantial neuroplasticity when provided with proper stimulation.

We expected that the prevalence of cerebral palsy would decrease in the education group, but there were even more infants diagnosed with cerebral palsy in the education versus control group. We presume that this is because the education group had more infants with a lower gestational age and more severe brain abnormalities than the control group. Although parent education helped children to develop better, it could not decrease the rate of cerebral palsy. Like our findings, a previous systematic review also concluded that early intervention did not change the rate of cerebral palsy [4].

Since the mid-1980s, early developmental intervention programs have aimed to improve the development of preterm infants. However, not all of these interventions were effective. For instance, when a modified version of the Mother-Infant Transaction Program was evaluated, neonatal nurses provided education to parents for mutually satisfying interactions during seven sessions before hospital discharge and at 3, 14, 30, and 90 days after discharge [28]. Although this intervention reduced parental stress in the intervention group (n = 69), it did not show improved cognitive, motor, or behavioral outcomes in the children at 2 years compared to the control group (n = 67). Another study found no effect of the Parent Baby Interaction Program (PBIP) on infant development at CA 2 years in preterm infants (<32 weeks gestational age). PBIP, delivered by nurses in weekly, 1 h sessions starting from the first week after birth and continuing for up to six post-discharge sessions, involved activities across four areas: discursive (e.g., discussing infant development), tactile (e.g., handling), verbal (e.g., talking to the baby), and observational (e.g., identifying behavioral states and cues) [29].

However, numerous reports also highlight successful early rehabilitation programs. In 1986, Barrera et al. [30] conducted a randomized controlled trial involving preterm patients with a mean gestational age of 29 weeks. The researchers compared a parent–infant intervention group (n = 22, focusing on parent–infant relationships) and a developmental program group (n = 16, focusing on infant development) with a standard follow-up group (n = 21). These programs were offered with home visits lasting 1–2 h that were weekly for the first 3 months, followed by visits every 2 weeks for the subsequent 6 months, and monthly visits during the last quarter of the year. Occupational or speech therapists delivered the interventions. Preterm infants in either program showed better MDI scores on the BSID-I in the CA 16 months group than in the standard follow-up group. This effect was more pronounced in infants with low (<1500 g) versus higher birthweight (1500–2000 g). Although the content of the intervention was similar to ours, differences existed in the intervention location (home vs. clinic), form (individualized vs. group-based), period of intervention (first 3 months vs. 6 months), and the number of sessions (27 vs. 8 sessions). Another paper [4] reviewed early developmental intervention programs provided after hospital discharge for preterm infants. These interventions encompassed physiotherapy, occupational therapy, neurodevelopmental therapy, psychologic therapy, parent–infant relationship enhancement, infant development and stimulation, and education. Twenty-five studies were reviewed, revealing that early intervention programs for preterm infants positively influenced cognitive and motor outcomes during infancy, with a larger effect size on cognitive outcomes versus motor outcomes (0.32 vs. 0.10), which was consistent with our findings. Since cognitive improvements persist into preschool age [4]; the children included in our study may also benefit in the cognitive domain during their preschool years. A recent systematic review also emphasized the importance of intervention starting as soon as possible [31]. The study reviewed 16 systematic reviews and 27 randomized controlled trials, and demonstrated that early rehabilitation can benefit from the plasticity of developing systems.

The above studies show the importance of proper design and execution of early intervention programs to promote optimal rehabilitation effects. Our study protocol focused on positively affecting family factors and the home environment associated with child development. The attitudes and perspectives of parents towards child development may have changed positively after the group-based parent education program. Evidence suggests that early high-quality parent–infant relationships positively influence children’s cognitive and social development [8]. Another study showed that a parent-guided developmental intervention provided knowledge and psychologic support for parents, which led to improved neurodevelopmental outcomes of infants [9]. Group-based education could be an effective method for further educating parents. A study comparing group-based versus individual-based training showed that group therapy was as effective as individual therapy [32]. Group therapy can increase social interaction between infants, and the therapy can increase the sense of competence between parents, motivating them to further engage in child rehabilitation.

There are several limitations in this study. First, the number of enrolled infants may be insufficient. A total of 246 infants participated, and subgroup analyses had even smaller sample sizes. Second, there were numerous dropouts during follow-up, with some possibly due to accessibility issues (such as moving closer to a local hospital) or reaching normal development. Also, the COVID-19 pandemic, which began in 2020, may have hindered parents from bringing their children to the hospital, leading to additional follow-up losses. Third, the baseline characteristics of the two groups were not well-balanced due to the study’s retrospective cohort design with historic controls. In addition, we only evaluated infant development up to 24 months of age. Assessing longer-term development may be needed to accurately evaluate infant development and long-term disability. Moreover, the study used BSID-II, not Bayley-III, due to availability problems. Finally, we were unable to analyze family education level and socioeconomic status due to insufficient data.

## 5. Conclusions

The eight sessions of group-based early parent education in preterm infants improved development at CA 24 months, especially in infants born before 28 weeks gestational age and in infants with no or mild brain injury. For better developmental outcomes, group-based early parent education should be encouraged in preterm infants. Further studies with assessments of the long-term development may further establish the effects of an early education program.

## Figures and Tables

**Table 1:** Activities of the group-based parent education program.

	CA 0–3 Months	CA 3–6 Months
Physical therapy (3 sessions)	Physiologic flexion training	Eye and hand coordination training
	Midline orientation training	Rollover training
	Supine to side-lying posture training	Creeping training
	Vestibular stimulation training	Quadruped position training
	Caring	Sitting postural training
	Vojta treatment (reflex turning 1)	Ball exercise; trunk control
		Vojta treatment (reflex turning 2, reflex creeping)
Occupational therapy (1 session)	Eye contact	Reach and grasp
	Staring at and tracking toys	Finger feeding
	Promoting caregiver–child interaction	Look toward auditory stimulation
		Promoting caregiver–child interaction

Abbreviation: CA, corrected age.

**Table 2:** Baseline characteristics of preterm infants.

Variables	Education Group (n = 101)	Control Group (n = 145)	*p*-Value
Gestational age at birth			0.005 *
<28 weeks	39 (38.6)	30 (20.7)	
28 to <32 weeks	34 (33.7)	54 (37.2)	
32 to <37 weeks	28 (27.7)	61 (42.1)	
Birthweight, g	1287.9 ± 575.2	1415.5 ± 533.8	0.075
Sex (male: female)	53 (52.5): 48 (47.5)	71 (43.0): 74 (51.0)	0.590
Brain lesion (yes: no)	62 (61.4): 39 (38.6)	80 (55.2): 65 (44.8)	0.334
Brain lesion			0.011 *
No abnormal findings	39 (38.6)	65 (44.8)	
IVH grade 1 or 2	26 (25.7)	53 (26.6)	
IVH grade 3 or 4 or PVL	18 (17.8)	9 (6.2)	
Others **	18 (17.8)	18 (12.4)	
Delivery			0.985
Vaginal delivery	13 (12.9)	19 (13.1)	
Cesarean section	79 (78.2)	126 (86.9)	
Unknown	10 (10.0)	0 (0.0)	
Surfactant use	54 (53.5)	63 (43.4)	0.007 *
Steroid use	56 (55.4)	100 (69.0)	0.862
ROP	23 (22.8)	23 (15.9)	0.157
NEC	7 (6.9)	8 (5.5)	0.631
Seizures	5 (5.0)	4 (2.8)	0.342
Surgery	28 (27.7)	69 (47.6)	0.432
Genetic anomalies	3 (3.0)	9 (6.2)	0.237

Values are numbers (%) of patients or mean ± standard deviation. Abbreviations: IVH, intraventricular hemorrhage; NEC, necrotizing enterocolitis; PVL, periventricular leukomalacia; ROP, retinopathy of prematurity; * *p* < 0.05 by independent *t*-test, Mann–Whitney U-test or Pearson’s Chi-squared test. ** For example, stroke, ventriculomegaly, or tubulinopathy.

**Table 3:** Developmental outcomes in preterm infants in each group.

Variables	Education Group (n = 101)	Control Group (n = 145)	*p*-Value
AIMS (3 months)	7.5 ± 2.8 [55]	10.0 ± 3.0 [119]	0.000 *
AIMS (6 months)	13.6 ± 4.6 [91]	16.2 ± 4.2 [111]	0.000 *
AIMS (9 months)	37.9 ± 10.3 [83]	33.2 ± 10.5 [97]	0.003 *
AIMS (12 months)	52.2 ± 5.3 [79]	50.1 ± 8.3 [85]	0.055
BSID II-MDI (12 months)	93.6 ± 13.4 [84]	92.4 ± 15.7 [110]	0.593
BSID II-PDI (12 months)	85.4 ± 16.3 [84]	86.1 ± 15.5 [110]	0.772
BSID II-MDI (24 months)	92.2 ± 20.4 [60]	86.5 ± 21.0 [63]	0.131
BSID II-PDI (24 months)	86.1 ± 20.6 [60]	85.8 ± 17.8 [63]	0.929
SELSI-RLI (24 months)	91.1 ± 24.5 [40]	85.4 ± 17.6 [40]	0.232
SELSI-ELI (24 months)	79.3 ± 26.9 [40]	75.2 ± 18.1 [40]	0.430
Cerebral palsy, n (%)	29 (28.7)	10 (6.9)	0.000 *

Values are mean ± standard deviation, unless otherwise indicated. Numbers inside square brackets [] indicate the numbers of children who had the particular measurement. * *p* < 0.05 by independent *t*-test or Mann–Whitney U-test. Abbreviations: AIMS, Alberta Infant Motor Scale; BSID II, Bayley Scale of Infant Development II; ELI, Expressive Language Index; MDI, Mental Development Index; PDI, Psychomotor Development Index; RLI, Receptive Language Index; SELSI, Sequenced Language Scale for Infants.

## Data Availability

The data presented in this study are available on request from the corresponding author due to privacy reasons.

## Appendix A

**Table A1:** Developmental outcomes according to gestational age.

	GA < 28 Weeks			GA 28 to <32 Weeks			GA 32 to <37 Weeks		
Variables	Education Group (n = 39)	Control Group (n = 30)	*p*-Value	Education Group (n = 34)	Control Group (n = 54)	*p*-Value	Education Group (n = 28)	Control Group (n = 61)	*p*-Value
AIMS (3 months)	7.0 ± 3.2 [22]	8.9 ± 2.8 [24]	0.034 *	7.8 ± 2.7 [17]	10.3 ± 3.0 [47]	0.004 *	8.0 ± 2.3 [16]	10.1 ± 3.1 [48]	0.014 *
AIMS (6 months)	13.0 ± 5.2 [38]	15.3 ± 4.1 [27]	0.065	13.6 ± 4.0 [29]	17.0 ± 3.8 [43]	0.001 *	14.6 ± 4.4 [24]	16.0 ± 4.6 [41]	0.237
AIMS (9 months)	36.9 ± 9.8 [36]	29.0 ± 9.9 [21]	0.005 *	20.1 ± 10.6 [10]	17.5 ± 7.3 [26]	0.413	41.3 ± 9.5 [20]	32.6 ± 11.0 [36]	0.005 *
AIMS (12 months)	51.8 ± 4.9 [33]	47.1 ± 10.2 [20]	0.065	36.7 ± 11.3 [27]	35.9 ± 9.7 [40]	0.775	53.8 ± 4.4 [21]	50.2 ± 9.9 [27]	0.106
BSID II-MDI (12 months)	89.3 ± 14.0 [33]	83.1 ± 14.6 [27]	0.096	94.8 ± 13.7 [28]	94.6 ± 13.3 [46]	0.951	98.2 ± 10.7 [23]	96.6 ± 16.8 [37]	0.650
BSID II-PDI (12months)	81.3 ± 15.3 [33]	76.2 ± 14.1 [27]	0.189	85.9 ± 17.0 [28]	89.2 ± 11.5 [46]	0.356	90.8 ± 16.1 [23]	89.4 ± 17.8 [37]	0.759
BSID II-MDI (24 months)	90.6 ± 21.2 [25]	78.0 ± 20.7 [21]	0.048 *	86.0 ± 20.6 [21]	89.2 ± 16.5 [28]	0.548	104.4 ± 13.2 [14]	94.0 ± 26.3 [14]	0.200
BSID II-PDI (24 months)	84.1 ± 22.5 [25]	76.2 ± 17.8 [21]	0.204	82.9 ± 19.7 [21]	92.1 ± 14.9 [28]	0.069	94.6 ± 16.7 [14]	87.6 ± 17.7 [14]	0.293
SELSI-RLI (24 months)	88.13 ± 26.0 [15]	78.2 ± 21.6 [12]	0.300	86.5 ± 25.6 [15]	87.1 ± 13.5 [18]	0.947	102.6 ± 17.8 [10]	91.2 ± 17.3 [10]	0.162
SELSI-ELI (24 months)	76.0 ± 28.5 [15]	69.0 ± 17.3 [12]	0.442	74.4 ± 26.8 [15]	76.4 ± 15.7 [18]	0.790	91.6 ± 22.9 [10]	80.5 ± 22.3 [10]	0.287

Values are mean ± standard deviation. Numbers inside square brackets [] indicate the numbers of children who had the particular measurement. * *p* < 0.05 by independent *t*-test or Mann–Whitney U-test. Abbreviations: AIMS, Alberta Infant Motor Scale; BSID II, Bayley Scale of Infant Development II; ELI, Expressive Language Index; MDI, Mental Development Index; PDI, Psychomotor Development Index; RLI, Receptive Language Index; SELSI, Sequenced Language Scale for Infants.

**Table A2:** Developmental outcomes according to brain injury.

	Brain Injury (–)			IVH Grade 1 or 2			IVH Grade 3 or 4, or PVL		
Variables	Education Group (n = 39)	Control Group (n = 65)	*p*-Value	Education Group (n = 26)	Control Group (n = 53)	*p*-Value	Education Group (n = 18)	Control Group (n = 9)	*p*-Value
AIMS(3 months)	8.0 ± 2.4 [23]	11.1 ± 3.3 [54]	0.000 *	6.7 ± 3.2 [14]	8.9 ± 2.0 [43]	0.003 *	8.1 ± 3.5 [10]	9.9 ± 2.6 [8]	0.251
AIMS (6 months)	14.6 ± 4.2 [37]	17.1 ± 3.9 [47]	0.008 *	13.4 ± 3.7 [24]	15.2 ± 3.2 [43]	0.044 *	12.5 ± 5.2 [16]	13.7 ± 4.6 [8]	0.570
AIMS (9 months)	38.2 ± 10.5 [33]	35.3 ± 10.4 [36]	0.248	38.1 ± 10.0 [21]	32.4 ± 9.5 [42]	0.029 *	38.9 ± 10.2 [16]	30.7 ± 13.3 [6]	0.137
AIMS (12 months)	53.2 ± 5.0 [29]	50.8 ± 6.9 [31]	0.136	53.7 ± 3.2 [22]	50.6 ± 6.0 [37]	0.027 *	51.6 ± 3.9 [16]	45.4 ± 16.8 [5]	0.460
BSID II-MDI (12 months)	97.7 ± 7.6 [31]	98.0 ± 11.7 [40]	0.880	93.8 ± 15.6 [23]	91.8 ± 14.1 [49]	0.591	90.1 ± 17.5 [15]	78.9 ± 21.8 [7]	0.207
BSID II-PDI (12 months)	91.7 ± 11.8 [31]	92.6 ± 12.3 [40]	0.751	85.9 ± 18.1 [23]	84.6 ± 13.9 [49]	0.736	77.3 ± 15.1 [15]	77.6 ± 19.4 [7]	0.975
BSID II-MDI (24 months)	102.9 ± 1 5.0 [19]	93.5 ± 17.5 [21]	0.077	92.6 ± 19.7 [18]	81.1 ± 19.6 [28]	0.059	81.0 ± 23.1 [10]	97.8 ± 28.3 [6]	0.418
BSID II-PDI (24 months)	98.1 ± 12.9 [19]	91.1 ± 15.5 [21]	0.131	85.8 ± 22.8 [18]	83.6 ± 17.0 [28]	0.709	79.5 ± 21.9 [10]	83.2 ± 21.8 [6]	0.750
SELSI-RLI (24 months)	102.1 ± 21.2 [13]	87.1 ± 11.6 [14]	0.037 *	95.9 ± 17.2 [13]	85.3 ± 20.6 [15]	0.162	69.2 ± 28.8 [9]	93.9 ± 9.7 [3]	0.186
SELSI-ELI (24 months)	89.7 ± 28.7 [13]	80.5 ± 16.0 [14]	0.309	79.5 ± 21.1 [13]	71.3 ± 15.9 [15]	0.254	61.9 ± 28.4 [9]	94.0 ± 13.6 [3]	0.096

Values are mean ± standard deviation. Numbers inside square brackets [] indicate the numbers of children who had the particular measurement. * *p* < 0.05 by independent *t*-test or Mann–Whitney U-test. Abbreviations: AIMS, Alberta Infant Motor Scale; BSID II, Bayley Scale of Infant Development II; ELI, Expressive Language Index; IVH, intraventricular hemorrhage; MDI, Mental Development Index; PDI, Psychomotor Development Index; PVL, periventricular leukomalacia; RLI, Receptive Language Index; SELSI, Sequenced Language Scale for Infants.
